# The intracellular interplay between galectin-1 and FGF12 in the assembly of ribosome biogenesis complex

**DOI:** 10.1186/s12964-024-01558-1

**Published:** 2024-03-11

**Authors:** Aleksandra Gędaj, Aleksandra Chorążewska, Krzysztof Ciura, Radosław Karelus, Dominika Żukowska, Martyna Biaduń, Marta Kalka, Małgorzata Zakrzewska, Natalia Porębska, Łukasz Opaliński

**Affiliations:** Department of Protein Engineering, Faculty of Biotechnology, University of Wroclaw, Joliot-Curie 14a, Wroclaw, 50-383 Poland

**Keywords:** FHF, FGF12, Galectins, Secretion, Nucleolus, NOLC1, TCOF1

## Abstract

Galectins constitute a class of lectins that specifically interact with β-galactoside sugars in glycoconjugates and are implicated in diverse cellular processes, including transport, autophagy or signaling. Since most of the activity of galectins depends on their ability to bind sugar chains, galectins exert their functions mainly in the extracellular space or at the cell surface, which are microenvironments highly enriched in glycoconjugates. Galectins are also abundant inside cells, but their specific intracellular functions are largely unknown. Here we report that galectin-1, -3, -7 and -8 directly interact with the proteinaceous core of fibroblast growth factor 12 (FGF12) in the cytosol and in nucleus. We demonstrate that binding of galectin-1 to FGF12 in the cytosol blocks FGF12 secretion. Furthermore, we show that intracellular galectin-1 affects the assembly of FGF12-containing nuclear/nucleolar ribosome biogenesis complexes consisting of NOLC1 and TCOF1. Our data provide a new link between galectins and FGF proteins, revealing an unexpected glycosylation-independent intracellular interplay between these groups of proteins.

**Supplementary Information:**

The online version contains supplementary material available at 10.1186/s12964-024-01558-1.

## Background

Fibroblast growth factors (FGFs) and their receptors (FGFRs) constitute signaling hubs that regulate human cell and body homeostasis [1]. The twenty-two FGF proteins are divided into seven subfamilies [2]. One of these is the fibroblast growth factor homologous factors (FHFs): FGF11 (FHF3), FGF12 (FHF1), FGF13 (FHF2) and FGF14 (FHF4), whose members are widely expressed in various cell and tissue types, such as fibroblasts, cardiomyocytes, neurons and osteoclasts [3]. FGF11-14 proteins do not have the ER-targeting signal peptide and were therefore for a long time considered solely intracellular proteins [3]. However, recent studies have demonstrated that a small portion of the FGF11-14 pool is secreted in the unconventional manner, and when present outside cells, they can directly bind FGFRs, triggering anti-apoptotic signaling [4, 5].

The precise function of intracellular FGF11-14 is vague, but it is clear that these proteins are highly important, as their dysregulation is linked with severe nervous and cardiac disorders and cancer [6–11]. Most studies classify FGF11-14 as modulators of plasma membrane voltage-gated ion channels [11–20]. Although partially localized to the cytosol, where FGF11-14 may encounter plasma-membrane embedded ion channels, FGF11-14 are primarily found in the nucleus and nucleolus, implicating the presence of significant nuclear activity of FGF11-14 [21]. Indeed, recent studies have shown that FGF11-14 interact with several nuclear proteins, including the nucleolar and coiled-body phosphoprotein 1, NOLC1 (Nopp140) and treacle ribosome biogenesis factor 1, TCOF1 (Treacle) [6, 21]. NOLC1 is a natively unfolded scaffold protein that forms a complex with TCOF1, which in turn connects RNA polymerase I with ribosome-modifying enzymes to facilitate ribosome biogenesis [22–24]. Notably, TCOF1 mutations are behind the ribosomopathy observed as Treachers-Collins Syndrome [25, 26]. Of the FGF11-14 proteins, only FGF12 is capable of binding both NOLC1 and TCOF1, while FGF11, FGF13 and FGF14 interact only with TCOF1, implicating at least partial functional diversification among FHF members [21]. It is not known what determines the nucleolar localization of FGF12, but it is clear that this phenomenon is independent of FGF12 interaction with NOLC1 or TCOF1 [21]. Importantly, nucleolar FGF12 appears to play a central role in the assembly of the nucleolar NOLC1/TCOF1 complex, as FGF12 knock-down negatively affected the interaction between NOLC1 and TCOF1, suggesting a significant role for FGF12 and other FHF members in ribosome biogenesis [6, 21].

The vast majority of secreted FGFs and all FGFRs are N-glycosylated, and this modification affects their stability, adjusts the interaction network and modifies subcellular localization [27–31]. We have recently demonstrated that the extracellular galectins, a family of multivalent lectins, decode information stored in N-glycan chains attached to FGFRs on the cell surface and secreted FGFs to fine-tune FGF/FGFR signaling [31–37]. Galectins are also found inside the cell, where they interact with several proteins binding their proteinaceous cores, participating among the others in protein trafficking, signal transduction and apoptosis [38]. In light of the significant role of galectins in N-glycan-dependent FGF/FGFR signaling and in the search for regulators of nuclear FGF12, we tested the interplay between intracellular FGF12 and selected members of the galectin family.

## Methods

### Antibodies and reagents

The primary antibody directed against FGF12 (#PA5-67182) and FGF13 (#S235-22) was from Thermo Fisher Scientific (Waltham, MA, USA). The primary antibodies: anti-NOLC1 (#sc-374033), anti-TCOF1 (#sc-374536), anti-FGF12 (#sc-81947), anti-Myc (#sc-40) were from Santa Cruz (Dallas, TX, USA). The primary antibody anti-NOLC1 (#HPA037366) was from Sigma-Aldrich (St Louis, MO, USA). The primary anti-galectin-1 (#12936) and anti-galectin-3 (#87985) antibodies were from Cell Signaling (Danvers, MA, USA). The primary antibodies directed against galectin-7 (#ab206435) and galectin-8 (#ab109519) were from Abcam (Cambridge, UK). Horseradish peroxidase-conjugated secondary antibodies were from Jackson Immuno-Research (Cambridge, UK). The secondary anti-rabbit antibody conjugated to Alexa Fluor 594 (#A11037) was from Thermo Fisher Scientific. Pierce Anti c-myc Magnetic Beads (#88843) was from Thermo Fisher Scientific. siRNA against galectin-1 (#sc-35441) was from Santa Cruz. The non-targeting control siRNA (#D-001810-01-50) was ordered from Horizon Discovery (Waterbeach, UK). The NucBlue Reagent (Hoechst 33342) (#R37605) and HCS CellMask Stain Deep Red (#32721) were from Thermo Fisher Scientific.

### Cells

The human osteosarcoma (U2OS) cell line was obtained from American Type Culture Collection (ATCC, Manassas, VA, USA). Cells were cultured in Dulbecco’s Modified Eagle’s Medium (DMEM) (Biowest, Nauille, France) supplemented with 10% fetal bovine serum (FBS) (Thermo Fisher Scientific) and antibiotics (100 U/ml penicillin, 100 µg/ml streptomycin) (Thermo Fisher Scientific). Stably transfected cell line U2OS-FGF12-GFP.myc [21] was cultured under the same conditions as U2OS cells, with the addition of 1 mg/mL geneticin (BioShop, G-418). Cell lines were grown in a 5% CO_2_ atmosphere at 37 °C. Cells were seeded onto tissue culture plates one day before the experiments.

### Recombinant proteins

Expression and purification of recombinant FGF12 and galectin-1,-3,-7,-8 were performed as described previously [4, 32].

### siRNA transfection

siRNA transfections were performed with DharmaFECT Transfection Reagents (Horizon, Cambridge, UK) according to the manufacturer’s instructions. Cells were transfected with 50 nM siRNA against galectin-1 or 50 nM non-targeting siRNA as a control. After 24 h, the transfection medium was replaced with the complete medium. Cells were incubated in a 5% CO_2_ atmosphere at 37 °C for another 24 h.

### Proximity ligation assay (PLA)

To analyze the interaction between studied proteins, Duolink® In Situ Fluorescence Protocol was used (Sigma-Aldrich). U2OS-FGF12-GFP.myc cells were fixed with 4% paraformaldehyde and permeabilized with 0.1% Triton in PBS. Cells were then incubated with appropriate antibodies and treated according to the manufacturer’s protocols. Cell nuclei were stained with NucBlue Live dye. Cytoplasm was stained with HCS Cell Mask Deep Red dye.

### Immunofluorescence staining

To analyze the co-localization of FGF12-GFP.myc and galectin-1,-3,-7 and 8, U2OS-FGF12-GFP.myc cells were fixed with 4% paraformaldehyde and permeabilized with 0.1% Triton in PBS. Cells were then stained with rabbit anti-galectin-1,-3,-7 and -8 primary antibodies and Alexa Fluor 594-conjugated anti-rabbit secondary antibody. Cell nuclei were stained with NucBlue Live dye.

### Fluorescence microscopy

Wide-field fluorescence microscopy was carried out using a Zeiss Axio Observer Z1 fluorescence microscope (Zeiss, Oberkochen, Germany) as described previously [21]. At least three independent experiments were carried out and at least 50 cells were used for quantification per experiment. Confocal fluorescence microscopy measurements were carried out using Opera Phenix Plus High-Content Screening system (Perkin Elmer, Waltham, MA, USA). Fixed cells were imaged in confocal mode using 63 × Water, NA 1.15 objective with binning 2 using two peaks autofocus. Images were performed using 2160 × 2160 px Camera ROI, 37 fields per well, with 8–10 Z-stacks per field at 0.5-μm interval to ensure comprehensive imaging of the cell. The Harmony High-Content Imaging and Analysis Software (version 5.1; Perkin Elmer, Waltham, MA, USA) was used for image acquisition and analysis. Number of cells was determined using the DAPI signal, which enables nuclei detection and the Cell Mask Deep Redd signal, which enables cytoplasm detection. Number of spots was determined based on Alexa Fluor 594 signal. Images were assembled in Illustrator (Adobe) with only linear adjustments of contrast and brightness.

### SPR measurements

Surface plasmon resonance (SPR) measurements were performed using the Biacore 3000 instrument (GE Healthcare) at 25°C. The FGF12 fused with the His-tag (in 10 mM sodium acetate, pH 6.0) was immobilized on the surface of a CM4 (low density) sensor chip (GE Healthcare) at approximately 600 RU, using an amine coupling protocol. To determine the kinetic constants of the interaction between galectin-1, -3, -7, -8 and FGF12, measurements were performed in HBS with 0.05% Tween 20, 0.05% BSA, 0.02%, NaN3, pH 7.4. A dilution set of galectins-1,-3,-7,-8 at concentrations ranging from 0.25 μM to 4 μM was injected at a flow rate of 30 μL/min. To regenerate the sensor, 2.5 M NaCl and 10 mM NaOH were applied between the injections. The data obtained were analyzed using BIA evaluation 4.1 software (GE Healthcare). The equilibrium dissociation constants (KD) were calculated from fitted saturation binding curves.

### Analysis of galectin-1 impact on FGF12 secretion

After galecin-1 silencing, FGF12 secretion analysis was performed in U2OS-FGF12-GFP.myc cells at 42°C, as previously described [5]. Media samples and cell lysates were analyzed by SDS-PAGE and western blotting with anti-FGF13 antibody, which is directed against peptide (AAAIASSLIRQKRQARE) present in FGF13 and FGF12. The amount of detected protein was quantified using ImageLab Software. Three independent experiments were quantified.

## Results and discussion

### Galectin-1, -3, -7 and -8 interact with FGF12 in the nucleus

**Fig. 1:**
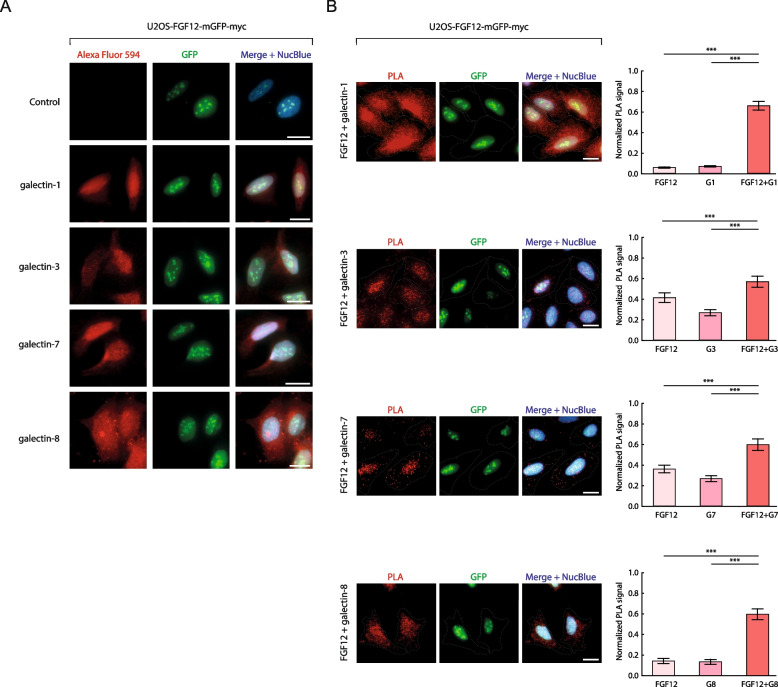
Galectins interacts with FGF12. **A** Co-localization of FGF12-GFP.myc with intracellular galectins. To analyze the co-localization of FGF12-GFP.myc with galectin-1,-3,-7 and 8, U2OS-FGF12-GFP.myc cells were fixed, permeabilized and stained with appropriate primary antibodies and Alexa Fluor 594-conjugated anti-rabbit secondary antibody. Cell nuclei were labeled with NucBlue and cells were analyzed by fluorescence microscopy. The scale bar represents 20 μm. **B** In situ proximity ligation assay (PLA) using mouse anti-myc antibody detecting FGF12-GFP.myc and rabbit antibodies directed against galectin-1,-3,-7, or 8 in U2OS-FGF12-GFP.myc cells. Cell nuclei were labeled with NucBlue and cells were analyzed by fluorescence microscopy. The dashed line indicates the cell area. The scale bar represents 20 μm. Data shown in the graphs are mean normalized PLA signal intensities ± SEM from three independent experiments. Statistical analyses were performed with Kruskal–Wallis H test (*p < 0.05; **p < 0.005 and ***p < 0.001)

We have recently demonstrated that ectopically expressed FGF12 fused to GFP is localized to the cytosol, nucleus and nucleolus in U2OS osteosarcoma cells [21]. To study the subcellular localization of four galectin members implicated in FGF/FGFR signaling: prototype galectin-1 and galectin-7, tandem-repeat galectin-8 and chimeric galectin-3 [32–34], we performed immunofluorescence microscopy experiments in U2OS cells stably producing FGF12-GFP.myc. As shown in Fig. 1A, all tested galectins were localized to the cytosol and nucleus. Importantly, none of the tested galectins displayed a clear-cut accumulation in FGF12-positive nuclear puncta characteristic for nucleoli (Fig. 1A).

Using proximity ligation assay (PLA), we tested whether intracellular galectins are capable of interacting with FGF12. As demonstrated in Fig. 1B, we detected significant PLA signals for all tested galectins and FGF12, with the strongest signal measured for the galectin-1/FGF12 pair. Importantly, for all galectins studied, the PLA signal was predominantly localized to the nucleus (Fig. 1B). In the case of galectin-1, we also observed strong PLA signals in the cytosol (Fig. 1B). These data indicate that galectin-1, -3, -7 and -8 form intracellular complexes with FGF12, and these complexes are mainly present in the nucleus.

### Galectins directly bind the proteinaceous core of FGF12

**Fig. 2:**
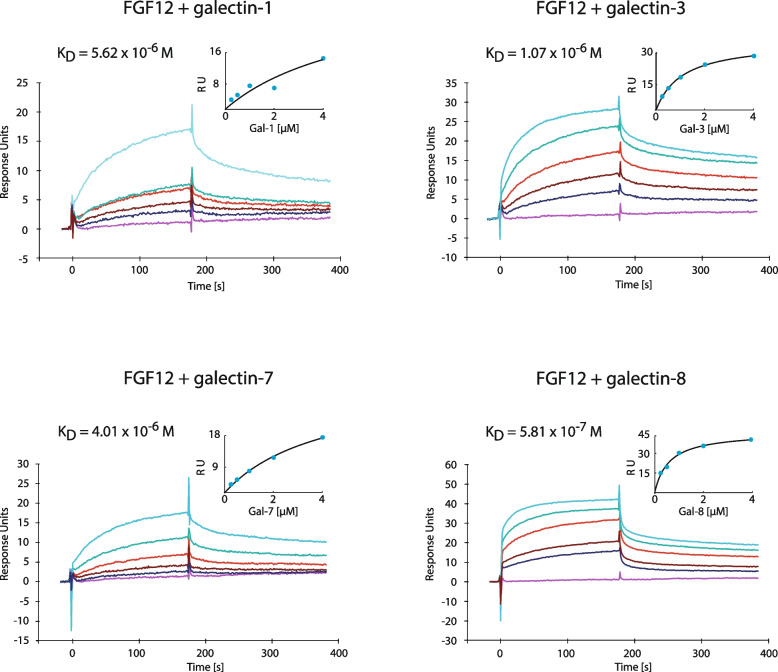
Kinetics of FGF12 interaction with galectin-1, -3, -7 and 8 assessed with SPR. Galectin-1, -3, -7, and -8 at the concentrations ranging from 0.25 μM to 4 μM were injected on CM4 sensor surface with FGF12 immobilized at low density (600 RU). Equilibrium dissociation constants (KD) were calculated from saturation binding curve

Since PLA experiments cannot accurately distinguish a direct interaction from an indirect one, we produced recombinant galectin-1, -3, -7 and -8 and FGF12 in a bacterial expression system and used the resulting proteins to measure intermolecular interactions using surface plasmon resonance (SPR). SPR experiments revealed specific and direct interactions between all galectins tested and FGF12 (Fig. 2). The measured affinities (K_D_) of galectins for FGF12 were in the micromolar (galectin -1, -3 and -7) or submicromolar range (galectin -8) (Fig. 2). Since in SPR assays we used recombinant galectins and FGF12 of bacterial origin incapable of glycosylation, our data suggest that the tested galectins interact directly with the proteinaceous core of FGF12. Interestingly, the affinities of galectins for FGF12 are in a similar range to the values obtained for galectin/N-glycosylated FGFs pairs [32]. All these data suggest that galectin-1, -3, -7 and -8 directly interact with FGF12.

### Galectin-1 is a negative regulator of FGF12 secretion

**Fig. 3:**
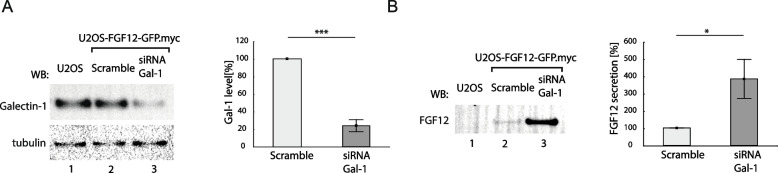
Role of galectin-1 in regulation of FGF12 secretion **A** Galectin-1 knock-down in U2OS-FGF12-GFP.myc cells. Western blotting analysis of cell lysates of U2OS-FGF12-GFP.myc cells treated with siRNA against galectin-1 and scramble siRNA as a control. The amount of detected galectin-1 was quantified using densitometry measurements with ImageLab Software (right panel). Mean values from three independent experiments ± SD are shown. Statistical analyses were performed with Student’s t-test (*p < 0.05; **p < 0.005 and ***p < 0.001). **B** Impact of galectin-1 on FGF12 secretion. U20S and U2OS-FGF12-GFP.myc cells after galectin-1 knock-down were serum-starved at 37°C for 24 h and, after medium exchange, incubated at 42°C for 2 h. The medium from above the cells was collected, centrifuged and incubated with anti-myc-tag magnetic beads. 50% of eluted samples were loaded onto SDS-PAGE gels and analyzed by western blotting. FGF12 protein was detected with anti-FGF13 antibody, recognizing all proteins belonging to FHF family. The amount of detected protein was quantified using densitometry measurements in ImageLab Software (right panel). Mean values ± SD from three independent experiments are shown. Statistical analyses were performed with Student’s t-test (*p < 0.05; **p < 0.005 and ***p < 0.001)

In further studies, we decided to focus on the intracellular interplay between galectin-1 and FGF12, since among the galectins tested, we were able to effectively knock-down only galectin-1 in U2OS-FGF12-GFP.myc cells (Fig. 3A). We have recently reported that FGF12-GFP.myc is secreted by U2OS cells [5]. The efficiency of unconventional FGF12 secretion was increased by elevated temperature, but the molecular mechanism of FGF12 release is still unknown [5]. To study whether the intracellular interaction between galectin-1 and FGF12 might affect FGF12 secretion, we analyzed the amounts of FGF12-GFP.myc found in the cell culture media upon galectin-1 knock-down with siRNA. As shown in Fig. 3B, depletion of U2OS-FGF12-GFP.myc cells of galectin-1 resulted in significantly increased level of extracellular FGF12-GFP.myc. Based on these findings, we hypothesize that highly abundant galectin-1 forms a stable complex with FGF12 in the cytosol and nucleus, which impedes FGF12 translocation through the plasma membrane.

### Galectin-1 constitutes a novel modulator of the FGF12/NOLC1/TCOF1 complex

**Fig. 4:**
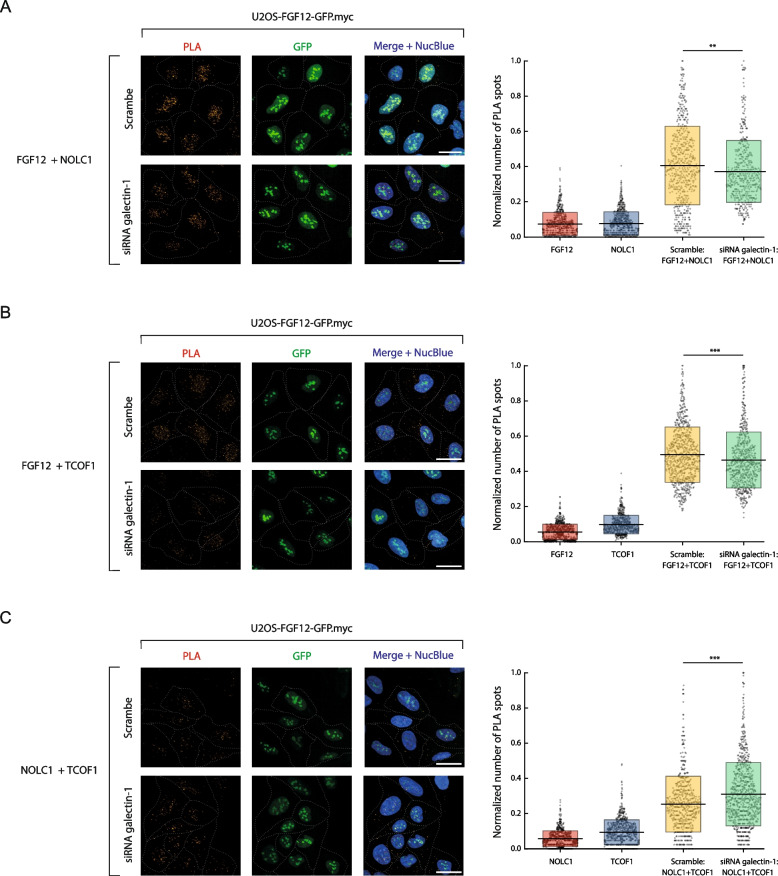
Galectin-1 modulate formation of FGF12/NOLC1/TCOF1 complexes. PLA-based analysis of galectin-1 impact on FGF12/NOLC1 (**A**), FGF12/TCOF1 (**B**), NOLC1/TCOF1 (**C**) interaction. Confocal images of in situ PLA using anti-FGF12, anti-NOLC1 and anti-TCOF1 antibodies in U2OS-FGF12-GFP.myc cells upon galectin-1 knock-down. Cell nuclei were labeled with NucBlue Live. The dashed line indicates the cell area. The scale bar represents 50 μm. Data shown in the graphs are normalized number of PLA spots in the cell. Single dot represents number of PLA spots recorded in individual cell. Boxes indicate mean ± SEM and lines represents mean from three independent experiments. Statistical analyses were performed with Mann–Whitney U test (*p < 0.05; **p < 0.005 and ***p < 0.001)

Previous studies have shown that nucleolar FGF12 interacts with NOLC1 and TCOF1 and that silencing of FGF12 results in a strongly reduced interaction between NOLC1 and TCOF1 [21]. These data imply that FGF12 may act as a molecular bridge physically linking NOLC1 to TCOF1. Using PLA in conjunction with the high content quantitative confocal microscopy, we studied the involvement of galectin-1 in the interaction of FGF12 with NOLC1 and TCOF1 [21]. To this end, we downregulated galectin-1 with siRNA and observed a significantly reduced interaction between FGF12 and NOLC1, and between FGF12 and TCOF1 (Fig. 4A and B). Interestingly, silencing of galectin-1 strongly enhanced the binding of NOLC1 to TCOF1 (Fig. 4C). These data implicate that galectin-1 directly binds FGF12 and promotes the interaction between FGF12 and NOLC1, and FGF12 and TCOF1. On the other hand, galectin-1 negatively regulates complex formation between NOLC1 and TCOF1, which is facilitated by FGF12.

## Conclusions

**Fig. 5:**
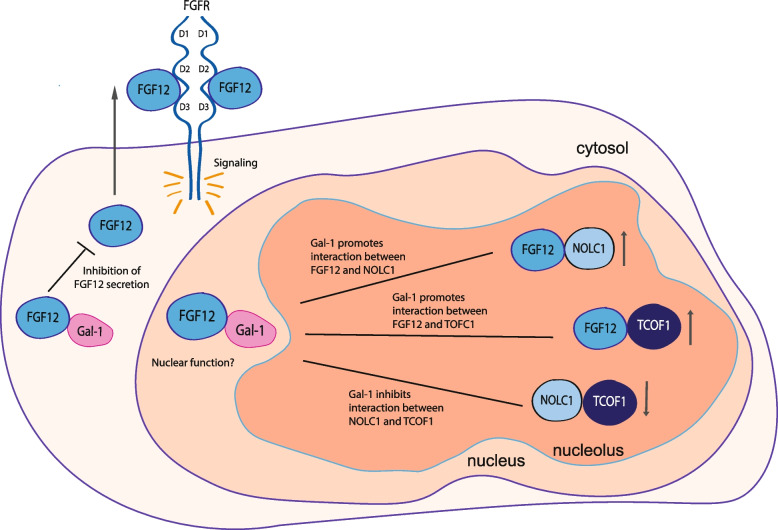
Hypothetical model of interplay between galectin-1, FGF12, NOLC1, TCOF1. Galectin-1 directly interacts with FGF12 in the cytosol and nucleus. Direct binding between these proteins inhibits the secretion of FGF12 (**A**). The nuclear function of FGF12—galectin-1 complexes remains unknown (**B**), but galectin-1 promotes the interaction of FGF12 with NOLC1 (**C**) and TCOF1 (**D**) and downregulates the binding of NOLC1 to TCOF1 (**E**)

In this work, we have provided a novel link between galectins and FGF/FGFR. In addition to the previously described N-glycosylation-dependent action of extracellular galectins on the glycosylated FGFRs and FGFs, our data suggest the presence of an intracellular interplay between galectins and FGF12 that is based on the direct interaction between proteinaceous cores of these protetins. Based on these data, we hypothesize that there is a dynamic interplay between galectin-1, FGF12, NOLC1 and TCOF1 that occurs in several subcellular compartments (Fig. 5). Galectin-1 directly interacts with the proteinaceous core of FGF12 to form galectin-1/FGF12 complexes in the cytosol and nucleus. By binding FGF12, galectin-1 captures FGF12 inside the cell and thus impedes the unconventional secretion of FGF12 (Fig. 5A). In the nucleus, galectin-1 affects the interplay between FGF12, NOLC1 and TCOF1. Galectin-1 promotes the assembly of FGF12/NOLC1 and FGF12/TCOF1 complexes (Fig. 5B and C) and inhibits the interaction between NOLC1 and TCOF1 (Fig. 5E). Further studies are needed to decipher the involvement of interplay between galectin-1, FGF12, NOLC1 and TCOF1 in nuclear homeostasis and function.

### Supplementary Information

**Supplementary Material 1.**

## Data Availability

All data and materials are available from the corresponding author upon a reasonable request.

## Abbreviations

BSA: Bovine serum albumin
DMEM: Dulbecco’s Modified Eagle’s Medium
FBS: Fetal bovine serum
FGF: Fibroblast growth factor
FGFR: Fibroblast growth factor receptor
FHF: Fibroblast homologous factor
Gal: Galectin
GFP: Green Fluorescent Protein
NOLC1: Nucleolar and coiled-body phosphoprotein 1
PLA: Proximity ligation assay
SPR: Surface Plasmon Resonance
TCOF1: Treacle ribosome biogenesis factor 1
