# Supplementary material for: The intracellular interplay between galectin-1 and FGF12 in the assembly of ribosome biogenesis complex
Source: Cell Commun Signal. 2024 Mar 11;22:175. doi: 10.1186/s12964-024-01558-1 (PMC10926643; doi:10.1186/s12964-024-01558-1)
Supplement: Supplementary file 1 — Supplementary Material 1. [file 12964_2024_1558_MOESM1_ESM.pdf]

Supplementary Information for

The intracellular interplay between galectin-1 and FGF12 in the assembly of ribosome biogenesis complex

Aleksandra Gędaj[1], Aleksandra Chorążewska[1], Krzysztof Ciura[1], Radosław Karelus[1], Dominika Żukowska[1], Martyna Biaduń[1], Marta Kalka[1], Małgorzata Zakrzewska[1], Natalia Porębska[1], and Łukasz Opaliński[1]*

[1]Department of Protein Engineering, Faculty of Biotechnology, University of Wroclaw, Joliot-Curie 14a, 50-383 Wroclaw, Poland

*Correspondence should be addressed to Ł.O (lukasz.opalinski@uwr.edu.pl)

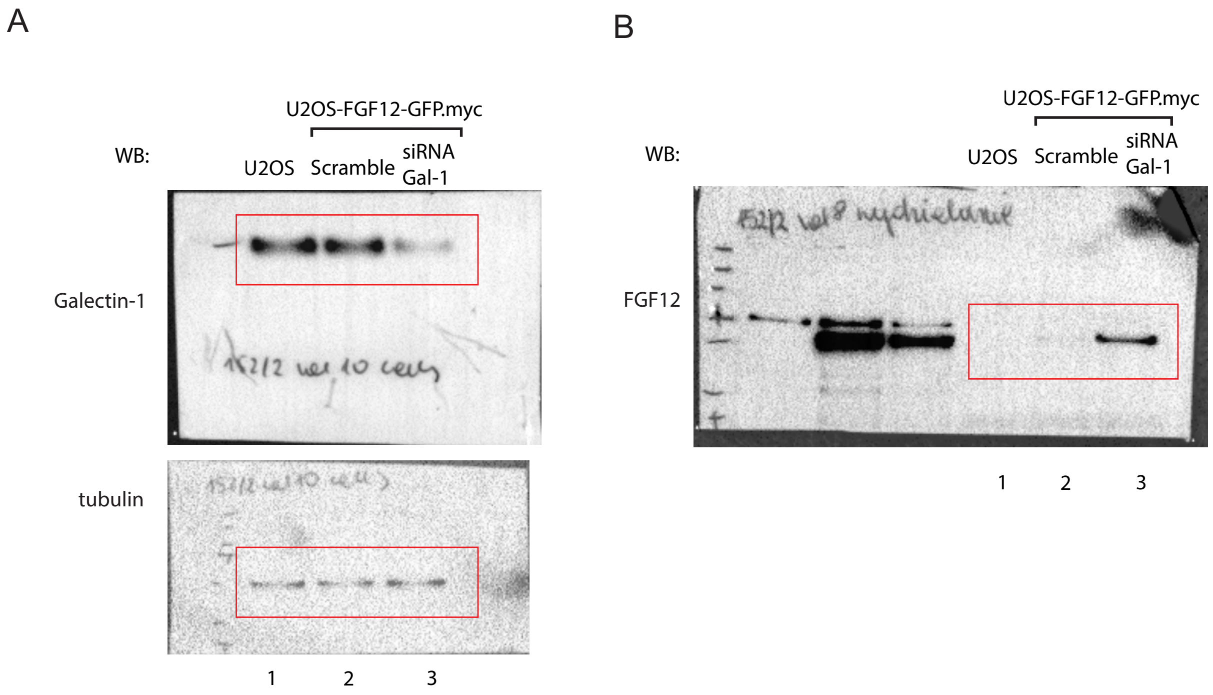

Uncropped blots from Figure 3
